# The Interaction Mechanism of Intramuscular Gene Delivery Materials with Cell Membranes

**DOI:** 10.3390/jfb14040219

**Published:** 2023-04-14

**Authors:** Zhanpeng Cui, Yang Jiao, Linyu Pu, Jianlin Chen, Ming Liu, James Zhenggui Tang, Gang Wang

**Affiliations:** 1 National Engineering Research Center for Biomaterials, College of Biomedical Engineering, Sichuan University, Chengdu 610064, China; 2 School of Materials and Chemistry, Southwest University of Science and Technology, Mianyang 621010, China; 3 School of Laboratory Medicine, Chengdu Medical College, Chengdu 610500, China; 4 Department of Medical Oncology/Gastric Cancer Center, West China Hospital, Sichuan University, Chengdu 610041, China; 5 Research Institute of Healthcare Science, Faculty of Science & Engineering, University of Wolverhampton, Wolverhampton WV1 1SB, UK

**Keywords:** molecular dynamics, potential of mean force (PMF), biomaterial, amphiphilic, triblock, phospholipid membrane, skeletal muscle

## Abstract

It has been confirmed that skeletal muscle cells have the capability to receive foreign plasmid DNA (pDNA) and express functional proteins. This provides a promisingly applicable strategy for safe, convenient, and economical gene therapy. However, intramuscular pDNA delivery efficiency was not high enough for most therapeutic purposes. Some non-viral biomaterials, especially several amphiphilic triblock copolymers, have been shown to significantly improve intramuscular gene delivery efficiency, but the detailed process and mechanism are still not well understood. In this study, the molecular dynamics simulation method was applied to investigate the structure and energy changes of the material molecules, the cell membrane, and the DNA molecules at the atomic and molecular levels. From the results, the interaction process and mechanism of the material molecules with the cell membrane were revealed, and more importantly, the simulation results almost completely matched the previous experimental results. This study may help us design and optimize better intramuscular gene delivery materials for clinical applications.

## 1. Introduction

In 1990, Wolff et al. injected naked plasmid (pDNA) into the skeletal muscles of mice and found that skeletal muscle cells could not only take up the pDNA but also express the corresponding protein [1]. This discovery opened a new direction for gene therapy. Although successful trials were acquired in the treatment of lower extremity ischemia [2], further applications, such as the treatment of cancers and diabetes, struggled to achieve satisfactory outcomes since the expression levels of functional proteins were not high enough for therapeutic purpose, which was mainly due to the extremely low efficiency of pDNA delivery into skeletal muscle cells [3,4]. Viral vectors have high gene delivery efficiency, but they are prone to trigger inflammatory responses and immunological reactions, as well as posing a risk of oncogenesis [5,6,7,8]. It was reported that an overdose injection of viral vector triggered an excessively strong inflammatory response, and eventually resulted in the death of the patient [9]. Therefore, non-viral gene carriers were anticipated to play an important role in the transfer of pDNA into skeletal muscle cells. In 1996, Mumper et al. mixed polyvinyl pyrrolidone (PVP) and polyvinyl alcohol (PVA) with pDNA, injected the mixture into the tibialis anterior (TA) muscles of mice and acquired an improved expression level of exogenous genes, which was about 10 times that achieved by pure pDNA injection alone [10]. This finding confirmed that pDNA delivery efficiency to skeletal muscles could be improved with the help of non-viral materials, and these materials were safer than viral vectors.

Non-viral gene delivery materials have been developed thereafter. Several new materials have been reported, such as PEG-Plys block copolymer [11], PEG-conjugated dendritic molecule PAMAM [12], and triblock copolymer PEG-PLGA-PEG [13]. Later, as commonly used excipients, Pluronic compounds came of interest to researchers. Pluronic compounds have an amphiphilic hydrophilic-hydrophobic-hydrophilic or hydrophobic-hydrophilic-hydrophobic triblock structure. It has been reported that Pluronic L64, P85, F127, P104, and SP1017 were able to improve the efficiency of gene transfer into skeletal muscles [14,15,16,17]. In our previous studies, Pluronic L64 was shown to be one of the most efficient and stable materials for pDNA delivery to skeletal muscles [2], and higher gene delivery efficiency was acquired when Pluronic L64 was combined with a low-voltage electric field [18,19], and was even high enough for therapeutic trials on tumors and diabetes [20,21]. Further, inspired by the similarity of amphiphilic structure between Pluronic L64 and cell membranes, amphiphilic triblock copolymers of LPL [22], rL_2_PL_2_, and rL_3_PL_3_ were designed and synthesized [23]. The results showed that LPL and rL_2_PL_2_ had better performance than Pluronic L64, evident from intramuscular gene delivery/expression efficiency and biocompatibility. Based on these studies, it can be concluded that amphiphilic triblock copolymers are able to disturb the structural stability of cytomembranes, leading to increased permeability, and the electric neutrality of the material molecules can prevent them from being trapped in the extracellular matrix of skeletal muscle tissues [24]. Therefore, electroneutral amphiphilic molecules with both “hydrophilic-hydrophobic-hydrophilic” and “hydrophobic-hydrophilic-hydrophobic” structures are ideal models for molecule design to optimize intramuscular gene delivery materials. However, the materials of LPL, rL_2_PL_2_, and rL_3_PL_3_ were all designed based on bionics, and the interaction mechanism of the materials with cytomembranes was only revealed at the cellular level [25]. To optimize the molecule design, it is necessary to understand the interaction process and mechanism between the materials and cell membranes at the molecular or even atomic scale.

In the early 1980s, molecular dynamics (MD) simulation was first applied to study biological systems [26]. With the development of computer technology, MD simulation has been applied to many studies on phospholipid membranes, such as the damage of graphene on cell membranes [27], the damage of graphene oxide on red blood cell membranes [28], the mechanism of action of cell-penetrating peptides [29], and the mechanism of cell membrane toxicity of petroleum oil droplets [30]. Therefore, MD simulation provides a novel tool for this kind of research. MD simulation is based on Newtonian mechanics, and a large number of theoretical calculations could be used to describe the interaction between molecules to obtain the mechanism of action at the atomic scale [31,32]. Furthermore, the conformations produced in MD simulation can be displayed in three dimensions by visualization software, which makes the outputs of MD more intuitive and easier to understand [33].

Compared with traditional experimental methods, MD simulation can be used to study the interaction between molecules in a system by establishing an appropriate model. Consequently, it can effectively shorten the experimental period, improve experimental efficiency, and reduce the consumption of experimental resources. From the perspectives of gene delivery materials, the data files obtained by MD simulation can be used to reveal the detailed interaction process among material molecules, cell membranes, and DNA, and to further explore the interaction mechanisms by means of structure and energy analysis.

Although there were many literature reports on the MD simulation of phospholipid membranes [34,35,36,37,38], few reports were found on the MD simulation regarding the interaction of gene delivery materials, especially amphiphilic triblocks, with cell membranes, and many conjectures were still derived from experience and assumptions [2,22,23,29,39,40,41,42]. Therefore, we will use MD simulation, taking the four previously reported materials L64, LPL, rL_2_PL_2_, and rL_3_PL_3_ as samples, to explore the mechanisms of their interactions with cell membranes by dynamically analyzing the intermolecular interactions and energy changes. It is anticipated that understanding the interaction process and mechanism at molecular and atomic levels will provide new inspiration for designing better materials for intramuscular gene delivery.

## 2. Computational Details

### 2.1. The Force Field Selection

The Charmm force field was selected for all simulations in this study. The force field version is Charmm36, which is an all-atom force field and can be used to study the interaction among nucleic acids, proteins, lipids, and other molecules [43,44]. The water model is TIP3P [45], which is suitable for the CHARMM force field.

### 2.2. The MD Simulation Software

The molecular dynamics simulation software used in this study was Gromacs 2018.4 [46,47,48]. The visualization software was VMD 1.9.3 [49].

### 2.3. Modeling of Material Molecules

In this study, four kinds of gene delivery polymer molecules, L64 and LPL (https://pubs.acs.org/doi/10.1021/am503808b, accessed on 15 August 2014), and rL_2_PL_2_ and rL_3_PL_3_ (https://pubs.acs.org/doi/10.1021/acsami.6b02592, accessed on 16 May 2016) were selected as the experimental objects. These four molecules are pure polymers. The structures of the molecules used in this paper are based on the actual experimental data from the previous studies of Liu S et al. [18] and Pu L et al. [22,23]. The experimental parameters of the gene delivery polymers are shown in Table 1.

**Figure 1:**
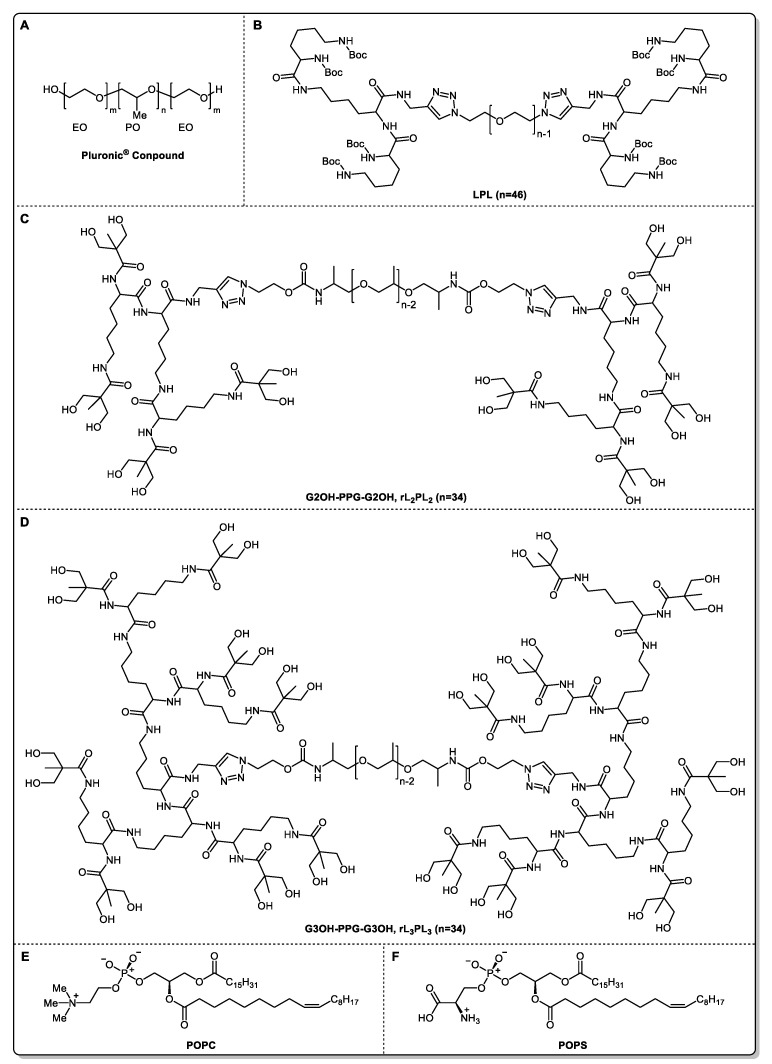
Structures of various gene delivery polymers: (**A**) Pluronic L64; (**B**) LPL; (**C**) rL_2_PL_2_; (**D**) rL_3_PL_3_; and cell membrane molecules: (**E**) POPC; (**F**) POPS.

The molecular structures are shown in Figure 1. The force field parameter files of the materials were obtained from the website CGenFF [50,51,52]. The total charge of each molecule was 0.

NVT stands for the canonical ensemble, a pre-equilibration step in which there are three constant quantities, namely, constant number of particles, constant volume, and constant temperature.

NPT, an isothermal isobaric ensemble, is the constant temperature and constant pressure ensemble, which is also a pre-equilibration step that includes three constant quantities, namely, constant particle number, constant temperature, and constant pressure. This approach can make the particle arrangement in the system approximate the actual particle arrangement, thereby improving the fidelity of the model and turning the system into a stable state.

After modeling the four material molecules, the energy in a vacuum was minimized in a grid with V = 30.0 nm × 30.0 nm × 30.0 nm, and the convergence limit was set to 100.0 kJ/mol·nm. Then, the NPT simulation at 500.0 ps was performed, and the pre-equilibration end conformation was taken as the initial conformation for the subsequent formal simulation.

The DNA molecule used in this study consisted of 14 consecutive A-T base pairs and was built using Avogadro 1.2.0 software [53,54]. The charge of the DNA molecule model in the environment of normal saline was −28 e^−^. The force field parameter files of the DNA were obtained from the website CGenFF [50,51,52].

### 2.4. Establishment of Cell Membranes

The cytoplasmic membrane was selected as a cell membrane containing 240 lipid molecules. It is composed of POPC (Figure 1E) and POPS (Figure 1F). The phase transition temperatures of POPC and POPS were set to 271.0 K and 300.0 K [55,56], respectively. The number ratio of the two lipid molecules, POPC:POPS was set to 4:1. The cell membrane was established by the Charmm-GUI server [57,58,59]. The thickness of the phospholipid membrane was about 4.0 nm. The membrane was located in the center of the grid (9.0 nm × 9.0 nm × 16.0 nm). The surface of the phospholipid membrane was perpendicular to the *z*-axis. The concentration of NaCl outside the membrane was 0.15 M.

### 2.5. Molecular Dynamics Simulation Ideas

In this study, we established 21 systems. The information on lattice size and the number of water molecules is shown in Supplementary Materials Table S1.

The deuterium order parameter can be written as:

(1) $$S_{\mathrm{CD}}=\frac{3\cos^{2}\theta_{i}-1}{2}$$

where *θ_i_* is the angle between the vector connecting the i − 1 and i + 1 beads of the lipid tail and the bilayer normal z, and *S*_CD_ is the average of the two chains of the same lipids in the entire bilayer and the designated simulation time. This allows a more quantitative assessment of the order of the phospholipid’s molecular orientation in membrane phospholipids. In this system, the order parameter can reflect the orderly arrangement of phospholipid molecules in the phospholipid membrane to quantitatively reflect the disturbance degree of the material to the cell membrane.

After the modeling was completed, a pre-equilibrium simulation was performed. The steps included energy minimization, NVT, NPT1, and NPT2. NVT and NPT1 limited the position of the phospholipid bilayer in the *z*-axis direction and the position of the material molecule in the three-dimensional direction. When using the modeling command to fill water molecules, the spatial orientation of all water molecules is consistent, that is, the high orientation of water molecules is consistent. This orientation certainly does not conform to the real situation. Therefore, before the simulation starts, we need first to carry out a pre-equilibrium step, that is, during the pre-equilibrium period, limit the orientation of material molecules and phospholipid molecules, and only let water molecules move in an irregular manner to approach the true situation of the irregular arrangement of water molecules in the natural state. The force constant of position limitation was 1000.0 kJ/mol·nm^2^, which was used to balance the orientation of the water molecules. The energy minimization adopted the steepest descent method. The energy convergence limit was set at 100 kJ/mol·nm, and the maximum number of calculation steps for energy minimization was set at 100,000.

The temperature control algorithm of NVT used the Berendsen method [60]. The pre-equilibration temperature was 310.0 K, and the duration was 1.0 ns. The temperature and pressure control algorithms of NPT1 also used the Berendsen method. The temperature and pressure control were set to 310.0 K and 1.0 bar, respectively, and the duration was 2.0 ns. NPT2 canceled the position restriction of the phospholipid membrane on the basis of NPT1 and continued to equilibrate for 2.0 ns.

**Scheme 1:**
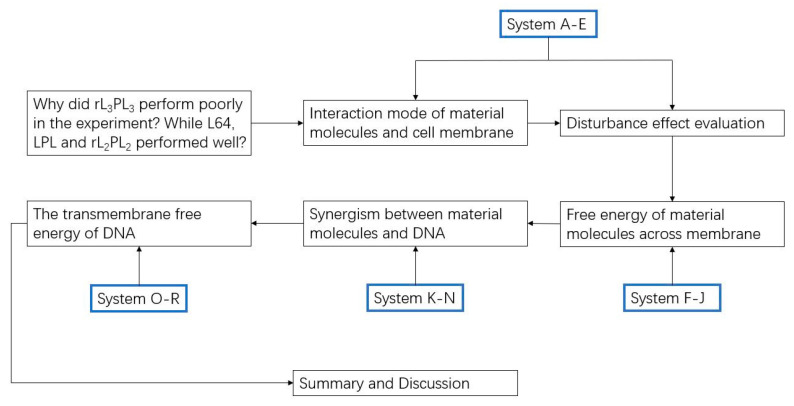
The process flow of the study simulations.

For formal MD simulations, the temperature control algorithm used the V-rescale method and the voltage control algorithm used the Parrinello–Rahman method [61,62]. The simulation duration was 100 ns. The simulation step size was 0.001 ps (corresponding to Systems A-E, K-N). In systems S-V, the simulation duration was 300 ns. The process flow of this study is shown in Scheme 1.:

The potential of mean force (PMF) refers to the potential energy obtained by integrating the mean force. When the direction of the average force is one-dimensional, the PMF can also reflect changes in free energy, thereby determining the spontaneity of intermolecular interactions [63,64,65]. (corresponding to Systems F-J, O-R).

The initial modeling legend is shown in Supplementary Materials Figure S1.

**Figure 2:**
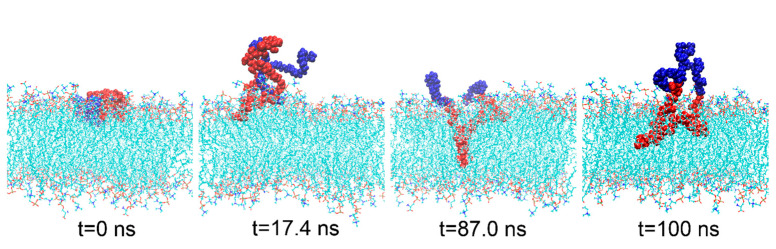
The process of L64 entering the phospholipid membrane. L64 was inserted about 1.0 nm into the phospholipid membrane (initial state). Red: hydrophobic segment; blue: hydrophilic segments.

**Figure 3:**
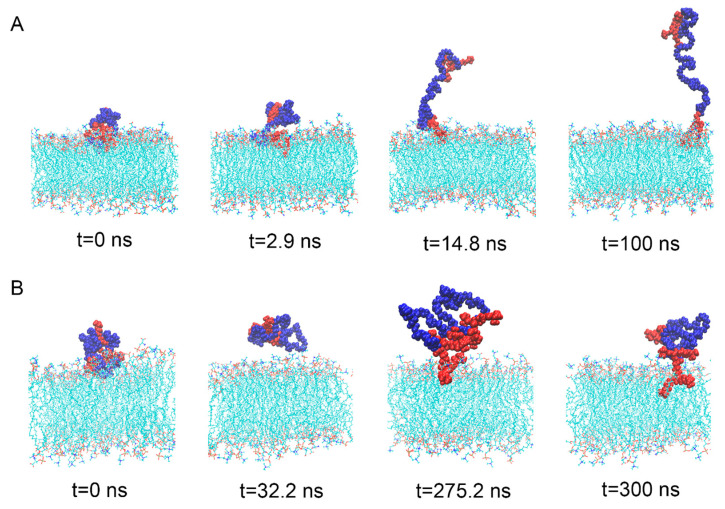
The process of LPL entering the phospholipid membrane. (**A**) LPL was inserted 1.0 nm into the phospholipid membrane (initial state). (**B**) LPL was out of the membrane (initial state). Red: hydrophobic segments; blue: hydrophilic segments.

**Figure 4:**
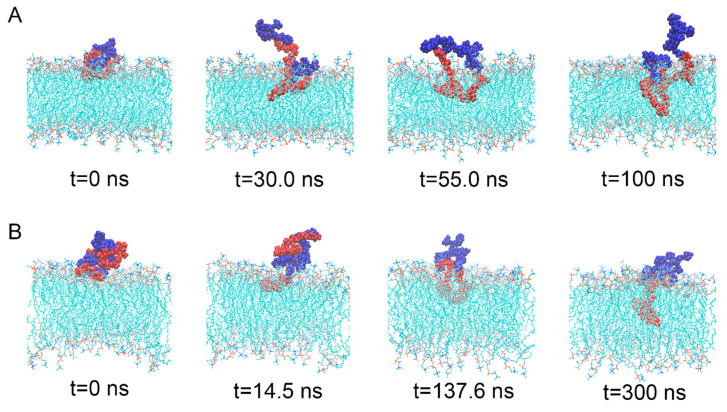
The process of rL_2_PL_2_ entering the phospholipid membrane. (**A**) rL_2_PL_2_ was inserted 1.0 nm into the phospholipid membrane (initial state). (**B**) rL_2_PL_2_ was out of the membrane (initial state). Red: hydrophobic segment; blue: hydrophilic segments.

**Figure 5:**
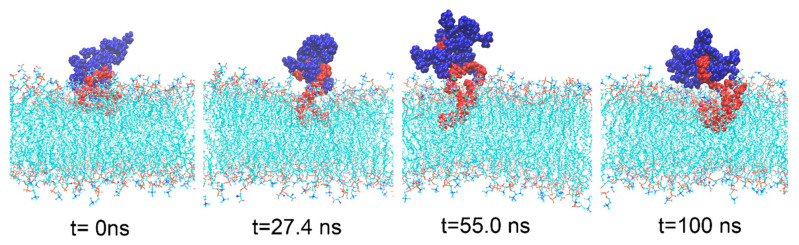
The process of rL_3_PL_3_ entering the phospholipid membrane. rL_3_PL_3_ was inserted 1.0 nm into the phospholipid membrane. Red: hydrophobic segment; blue: hydrophilic segments.

In systems A, B, D, and E, in order to study the disturbing effect of the four material molecules on the cell membrane, we inserted four material molecules into the cell membrane at 1.0 nm, created a phospholipid membrane disturbing environment (corresponding to Figure 2, Figure 3A, Figure 4A and Figure 5), each system was repeated three times independently. The configurations were chosen every 0.1 ns during the molecular dynamics simulations.

In system C, we put LPL directly into the center of the phospholipid membrane and observe its disturbing effect (corresponding to LPL in), each system was repeated three times independently. The configurations were chosen every 0.1 ns during the molecular dynamics simulations.

In system F-J, we place the four material molecules above the phospholipid membrane, and the distance between them and the centroid of the phospholipid membrane is 4.0 nm. This initial model is used to calculate the change in free energy of the four material molecules, and the calculation methods were PMF and WHAM. For the PMF simulation, we apply a constant force of 1000.0 kJ/mol·nm^2^ perpendicular to the phospholipid membrane and set its maximum traction speed as 0.01 nm/ps. The traction distance is 9.0 nm, so we set the sampling window to 0.20 nm, and the sampling window is 50, to ensure that there is enough intersection between the two adjacent windows. The pre-equilibration time of each window was 300.0 ps, and the relaxation time was 8.0 ns. After the relaxation of all windows was completed, the weighted distribution histogram was drawn using the WHAM module of Gromacs [66]. The change curve of PMF free energy was obtained, and each system was repeated twice independently.

In system K-N, we put the material molecules and DNA into a lattice separately, and the distance between the DNA and material molecules is 0.3 nm—close but without contact—and each system was repeated three times independently. The configurations were chosen every 0.1 ns during the molecular dynamics simulations. 

In system O-R, we put DNA and different phospholipid membranes into a system, where DNA molecules are placed above the phospholipid membrane. The distance from the centroid of the phospholipid membrane is 5.0 nm, and the double helix direction of DNA is parallel to the surface of the phospholipid membrane. This initial model is used to calculate the change in DNA transmembrane free energy, and the calculation methods were PMF and WHAM. For the PMF simulation, we apply a constant force of 1000.0 kJ/mol·nm^2^ perpendicular to the phospholipid membrane and set its maximum traction speed as 0.01 nm/ps. The traction distance is 10.0 nm, so we set the sampling window to 0.18 nm, and the sampling window is 60, to ensure that there is enough intersection between the two adjacent windows. The pre-equilibration time of each window was 300.0 ps, and the relaxation time was 8.0 ns. After the relaxation of all windows was completed, the weighted distribution histogram was drawn using the WHAM module of Gromacs [66], and each system was repeated twice independently.

In system S-U, we put L64, LPL, and rL_2_PL_2_ at 1.0 nm above the surface of the phospholipid membrane to observe how the material molecules enter the phospholipid membrane.

## 3. Results and Discussion

### 3.1. Interaction Process between Material Molecules and Phospholipid Membranes

In order to observe how the material molecules interacted with cell membranes, the material molecules were inserted about 1.0 nm into the membrane, and molecular dynamics simulation was performed for 100 ns. It can be found that when L64 was not repelled by the cell membrane, it flowed with the phospholipid membrane and gradually entered the centroid of the phospholipid membrane. The molecules became linearly arranged with time, and the hydrophobic segment flowed stably in the cell membrane while the hydrophilic end swung in the solvent (Figure 2).

When L64 molecules were located above the phospholipid membrane, they floated randomly outside the membrane and were not did not easily come into contact with the phospholipid membrane. In order to increase the probability of L64 entering the cell membrane, we set up a bilayer membranes model and put L64 molecules between the two membranes. In this system, L64 molecules were found to automatically enter the phospholipid membrane (Supplementary Materials Figure S2). At 139.2 ns, the hydrophobic part of L64 began to come into contact with the hydrophobic region of the membrane. Then, the L64 molecule gradually moved toward the center of the membrane, and the final conformation was consistent with that when L64 was inserted 1.0 nm into the phospholipid membrane. The overall extension of L64 became linear. Its hydrophobic segment flowed stably in the hydrophobic area of the phospholipid membrane, while the hydrophilic end swung randomly in the water. The results revealed that the entry of L64 molecules into the cell membrane mainly relied on the hydrophobic interaction.

As the LPL molecule has the “hydrophobic-hydrophilic-hydrophobic” structure, whereas molecules of L64, rL_2_PL_2_, and rL_3_PL_3_ are “hydrophilic-hydrophobic-hydrophilic” in arrangement, the LPL molecule was initially inserted 1.0 nm into the phospholipid membrane. About 14.8 ns later, one of the hydrophobic segments re-entered the water and floated there, while another hydrophobic segment end was still kept in the hydrophobic region of the membrane. This pattern was maintained until the end of the simulation (Figure 3A). In addition, we also observed how the LPL molecule gradually embedded in the phospholipid membrane when it was initially located outside the membrane (Figure 3B). At 275.2 ns, a hydrophobic segment of the LPL molecule protruded into the hydrophobic region of the membrane, forming a mosaic state. At 300 ns, the conformation was largely the same as that when LPL was inserted 1.0 nm into the membrane—namely, one hydrophobic segment was embedded in the membrane and the other floated irregularly in the water. The results revealed that the entry of the LPL molecule into the cell membrane also depended on the hydrophobic interaction, similar to the case with L64.

When rL_2_PL_2_ was inserted into the membrane, it showed a similar phenomenon to L64. From 55.0 ns, the hydrophobic segment of rL_2_PL_2_ was embedded in the membrane, and the hydrophilic ends floated on the membrane surface (Figure 4A). However, unlike L64, rL_2_PL_2_ molecules did not have a clear tendency to move to the center of the membrane but were embedded in a relatively fixed position on the membrane, indicating that rL_2_PL_2_ could interact with both the hydrophobic and hydrophilic regions of the phospholipid membranes. The hydrophilic ends of rL_2_PL_2_ were also in contact with the external aqueous solution. Meanwhile, the membrane entry process of rL_2_PL_2_ was also observed when rL_2_PL_2_ was put on the surface of the membrane (Figure 4B). Similar to L64, when the hydrophobic part of rL_2_PL_2_ contacted the hydrophobic region of the membrane, rL_2_PL_2_ could gradually enter the center of the membrane, until the hydrophobic part was inside and the hydrophilic ends were outside the membrane.

When the rL_3_PL_3_ molecule was inserted into the phospholipid membrane, the phenomenon was similar to that of the rL_2_PL_2_. But the difference was that rL_3_PL_3_ did not exhibit a mosaic state similar to that of rL_2_PL_2_ until 70.0 ns. The rL_3_PL_3_ molecule embedded in a shallower place of the membrane and its hydrophilic segments tended to be clumped together (Figure 5).

To sum up, no matter what material, as long as the hydrophobic part comes into contact with the hydrophobic region of the membrane, the material will enter the membrane spontaneously and will not be repelled from the cell membrane.

### 3.2. Transmembrane Free Energy Analysis of the Materials

**Figure 6:**
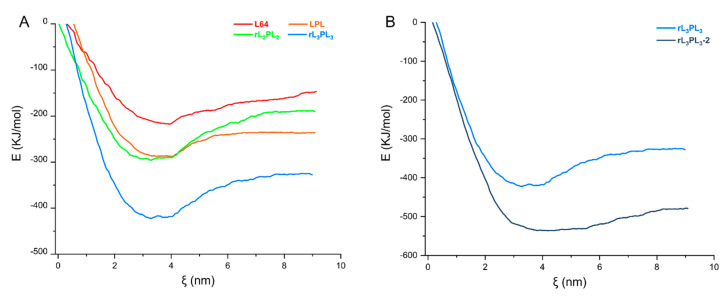
Changes in transmembrane free energy of the four molecules. The phospholipid membrane was located at coordinates 2.0–6.0 nm. (**A**) L64, LPL rL_2_PL_2_, and rL_3_PL_3_. (**B**) rL_3_PL_3_ and rL_3_PL_3_-2. rL3PL3: hydrophobic end of rL_3_PL_3_ facing the membrane; rL_3_PL_3_−2: hydrophilic ends of rL_3_PL_3_ facing the membrane.

In this step, the transmembrane free energy of the four materials was analyzed. After the attachment of the materials to the cell membrane, the free energy sharply decreased (Figure 6), indicating that the four materials could easily attach and enter the phospholipid membrane. In summary, the energy changes were rL_3_PL_3_ > rL_2_PL_2_ = LPL > L64. However, when the materials left the membrane, in all cases the energy slowly increased to a varying degree, implying that the four materials molecules all had a tendency to be detained in the center of the cell membrane, which may be due to electric interaction between the material and membrane. It is obvious that rL_2_PL_2_ caused a higher energy change than LPL while exiting the membrane, implying that the LPL molecule could exit the membrane more easily.

In summary, the four materials showed the same characteristics in that the free energy reduced while entering the membrane and increased while exiting the membrane. These results also explained that when the material molecules were inserted 1.0 nm into the membrane, they were not repelled but spontaneously moved toward the center of the membrane.

**Figure 7:**
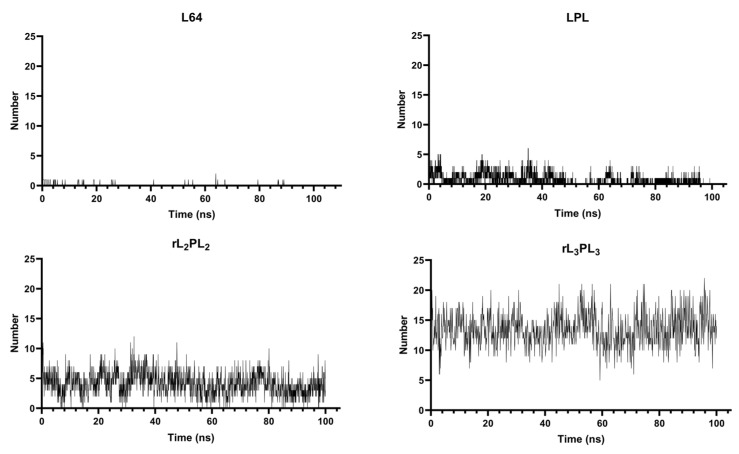
Analysis of the number of intramolecular hydrogen bonds.

We found that after entering the cell membrane, L64, LPL, and rL_2_PL_2_ would change from their initial shape to a linear or dumbbell shape, while rL_3_PL_3_ still kept the initial clustering structure (Figure 2, Figure 3, Figure 4 and Figure 5). Meanwhile, the final morphologies of the four molecules were also confirmed in the PMF process (Figure S3). According to the molecular structure, we speculated that the conformational change may be caused by intramolecular hydrogen bonds. To address this, we analyzed the number of intramolecular hydrogen bonds in the four molecules. The results showed that the rL_3_PL_3_ molecule contained about 13 intramolecular hydrogen bonds, while other materials had fewer than 10 bonds (Figure 7). This meant that rL_3_PL_3_ had stronger intramolecular interaction than the other molecules, so the rL_3_PL_3_ molecule could not easily undergo a conformational change but was inclined to keep its initial conformation. The existence of excessive intramolecular hydrogen bonds would reduce the flexibility of the conformational change of the molecule, thus finally weakening the disturbance effects. The results of hydrogen bond analysis were also consistent with our previous research, namely, rL_3_PL_3_ had the worst gene delivery results among the four molecules [2,22,23].

### 3.3. Parametric Analysis of Phospholipid Membranes

**Figure 8:**
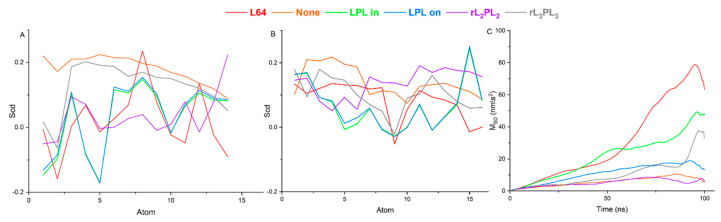
Parameter analysis of the phospholipid membrane after interaction with the four molecules respectively. LPL in: the entire LPL molecule embedded in the membrane. LPL on: half of the LPL embedded in the membrane. Lipid: the membrane alone, without any gene delivery molecule. (**A**) The deuterated order parameter of the sn-1 chain; (**B**) the deuterated order parameter of the sn-2 chain; (**C**) the lateral diffusion coefficient M_sd_ of the membrane. None: the membrane without any gene delivery molecule.

To further understand the mechanism of these four materials in gene delivery, we analyzed the changes in deuterium order parameters and the cell membrane transverse diffusion coefficient and compared the perturbation effects of the two final conformations of LPL on cell membranes. The results showed that L64, LPL, and rL_2_PL_2_ had potent disturbance effects on the cell membrane, but rL_3_PL_3_ had just a little (Figure 8A). This may be the reason why rL_3_PL_3_ did not function as well as other materials in previous intramuscular gene delivery studies [2,22,23]. At the same time, there was little difference in the disturbance effects of “LPL in” and “LPL on” (Figure 8A,B), indicating that the contact area with the phospholipid membrane did not affect the disturbance effects of LPL.

We found that the lateral diffusion coefficients (M_sd_) of these materials did not decrease. Even the lowest rL_2_PL_2_ group had the same lateral diffusion coefficient as the “None” group (Figure 8C). This indicated that when these materials were used for gene delivery, they would not affect the fluidity of the cell membrane. These results were considered to explain the reason why the materials had almost no cell membrane toxicity [2,22,23].

**Figure 9:**
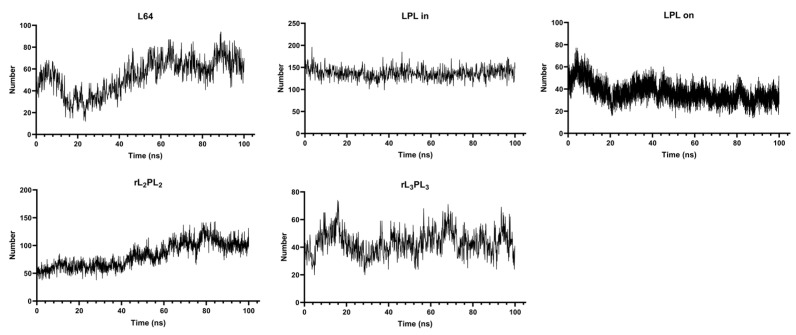
Atom numbers in the phospholipid membrane in contact with different material molecules. LPL in: the whole LPL molecule was embedded in the membrane. LPL on: LPL was only half embedded in the membrane.

In order to study the contact details between material molecules and cell membranes, we calculated the atom number in the phospholipid membrane in contact with the material molecules, which can reflect the contact area between the material molecules and the membrane. The results showed that for the “hydrophilic-hydrophobic-hydrophilic” molecules, the number of contacted atoms ran rL_2_PL_2_ > L64 > rL_3_PL_3_ (Figure 9). This was completely consistent with the disturbance effects (Figure 8A,B) and gene delivery efficiency data of the three molecules in our previous research [2,22,23]. Therefore, we found that for such materials, the number of contacted atoms between the material and the membrane could directly affect the disturbance effect and consequently, the intramuscular gene delivery efficiency. Conversely, as a “hydrophobic-hydrophilic-hydrophobic” triblock material, the “LPL on” had obviously fewer atomic contacts than “LPL in”, but both the “LPL on” and “LPL in” had the same disturbance effects (Figure 8A,B). This means that the delivery mechanism of LPL may be different from that of “hydrophilic-hydrophobic-hydrophilic” materials L64, rL_2_PL_2_, and rL_3_PL_3_.

### 3.4. The Interaction between DNA Molecule and Material Molecule

**Figure 10:**
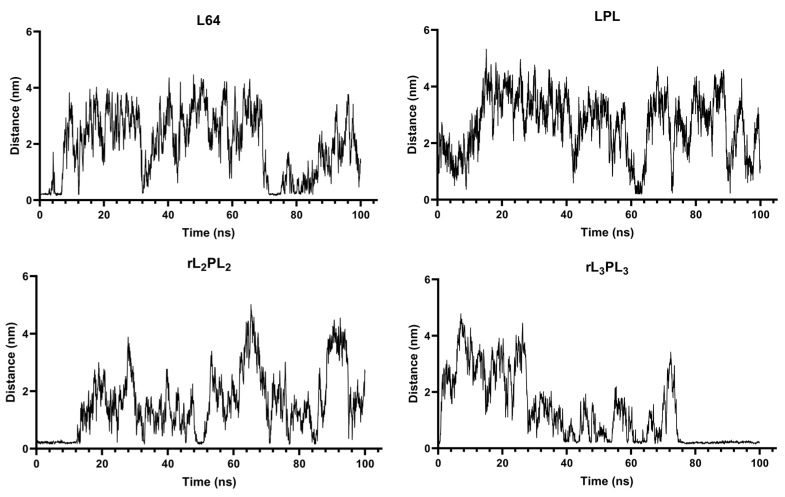
Minimum distance change between the DNA molecule and the material molecule.

To analyze the interaction between a DNA molecule and a material molecule, a model system was built. In this model, the initial centroid distance was 3.0 nm and the lattice was 14.0 nm × 8.0 nm × 8.0 nm. The same pre-equilibration steps and parameters were used for the material and the membrane, and the simulation time was 100.0 ns. According to the real-time minimum distance map, no contact was displayed between the material molecule and the DNA molecule for most of the duration of the simulation (Figure 10). Therefore, it could be concluded that there was basically no interaction between the materials and DNA. These results were consistent with previous gel retardation assays [2,22,23].

### 3.5. Transmembrane Free Energy of DNA Molecule

In our previous experimental studies, it was confirmed that in the presence of material molecules, the delivery efficiency of DNA was higher than that of DNA alone [2,22,23]. Here, a free energy analysis was performed to explore the mechanism for the improved gene delivery efficiency. In this step, material molecules were placed in the membrane to perturb the membrane in advance for 100.0 ns, and then the transmembrane free energy of DNA was calculated. Only the molecules of L64 and LPL were investigated in this simulation since they had opposite amphiphilic structures.

**Figure 11:**
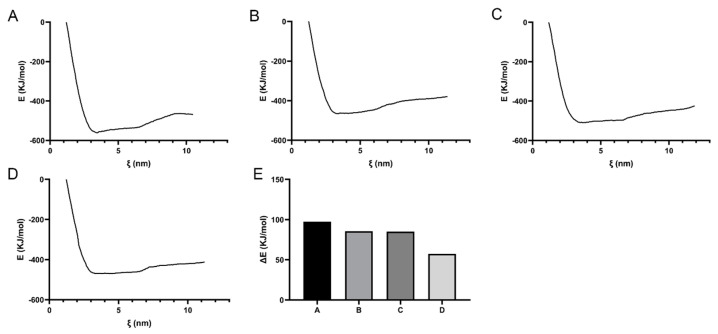
Changes in DNA transmembrane free energy. The phospholipid membrane was located at coordinates 4.0–8.0 nm. (**A**) across pure membranes; (**B**) across L64-perturbed membranes (DNAs did not encounter L64 molecules); (**C**) across LPL-perturbed membranes (DNA encountered LPL molecules); (**D**) across LPL-perturbed membranes (DNA did not encounter LPL molecules); (**E**) Changes in DNA transmembrane free energy of (**A**–**D**).

For cell membranes without material perturbation, the transmembrane energy barrier to DNA was 97.3 kJ/mol. After L64 perturbation, the transmembrane energy barrier decreased to 85.6 kJ/mol, and after LPL perturbation, it decreased to 57.2 kJ/mol. However, when DNA encountered the LPL molecule in the membrane, the energy barrier became 85.2 kJ/mol (Figure 11). The results showed that after LPL perturbed the cell membrane, the DNA transmembrane energy barrier would significantly decrease (97.3 kJ/mol to 57.2 kJ/mol), which consequently made it easier for DNA to penetrate the membrane. It was also clear that in terms of lowering the DNA transmembrane energy barrier, LPL was better than L64 (57.2 kJ/mol vs. 85.6 kJ/mol). The results were still consistent with previous experimental results regarding intramuscular gene delivery [22]. In addition, as the DNA transmembrane energy barrier would be negatively influenced while encountering the LPL molecules in the membrane (57.2 kJ/mol vs. 85.2 kJ/mol), it would be better to reduce the retention of material molecules in the membrane.

## 4. Discussion

Traditional cationic polymer gene delivery materials, such as polyethyleneimine (PEI), usually electrostatically interact with DNA molecules and condense them into a more compact structure, called, for example, the PEI-DNA complex. When DNA is compressed, it can be protected from being degraded by nucleases outside and inside target cells, and there is an interaction between the DNA and material molecules throughout the action process [67,68]. In contrast, the nonionic triblock copolymer materials we studied here showed almost no interaction with DNA during the entire interaction, and contact between them was absent most of the time (Figure 10). These characteristics have been proved by DNA retardation assays in previous studies [2,22,23]. Therefore, this kind of material and DNA act independently and successfully during the transmembrane process.

Based on the simulation results, the interaction between material molecules and DNA molecules, the process of material molecules contacting and entering the membrane, and the change in DNA transmembrane free energy were systematically explored and displayed. The results showed that the four material molecules all had a tendency to interact and enter the membrane, and to be embedded in the phospholipid membrane at a certain time (Figure 2, Figure 3, Figure 4 and Figure 5). The analysis of the free energy showed that this process was accompanied by a decrease in free energy, revealing that the materials could enter the cell membrane spontaneously (Figure 6). When the hydrophilic ends of the materials faced the phospholipid membrane, the free energy was reduced to a lower value (Figure 6B), so, it could be inferred that the hydrophilic end of a material molecule should first contact and enter the phospholipid membrane. Subsequently, both the “hydrophilic-hydrophobic-hydrophilic” and “hydrophobic-hydrophilic-hydrophobic” molecules utilized their hydrophobic ends to contact with the hydrophobic area of the membrane and then enter the membrane through the hydrophobic interaction (Figure 2, Figure 3, Figure 4 and Figure 5). When the distance between the material molecule and the mass center of the membrane was zero, the free energy reached its lowest value (Figure 6), which also demonstrated that the free energy change process is spontaneous and energy-independent.

For the “hydrophilic-hydrophobic-hydrophilic” copolymer molecules, including L64, rL_2_PL_2_, and rL_3_PL_3_, it was found that their perturbation effects on the cell membrane depended on the number of contacted phospholipid atoms (Figure 9). The number of phospholipid atoms contacted by the material molecules ran rL_2_PL_2_ > L64 > rL_3_PL_3_ (Figure 9). Previous experiments revealed that rL_2_PL_2_ had the best in vivo gene delivery and expression outcomes, but L64 had almost the same results as those of rL_3_PL_3_ [23]. This may be attributed to the following reasons. L64 almost has no intramolecular hydrogen bonds (Figure 7), so it can be fully stretched in the cell membrane and, therefore, has a large contact area. However, the molecular weight and size of L64 are relatively small, thus limiting its perturbation effect on the membrane. Conversely, rL_3_PL_3_ is the largest molecule, but it has more internal hydrogen bonds than the other molecules (Figure 7), which may limit the ability of the molecule to change its confirmation, thus reducing the contact with membrane atoms and weakening the perturbation effect on the membrane. Meanwhile, the large size of the rL_3_PL_3_ molecule can also make it more difficult for it to enter the cell membrane, thus remaining at a shallower position than rL_2_PL_2_ (Figure 5).

From the intramuscular gene delivery and expression experiments, we already knew that LPL was more effective than L64, and rL_2_PL_2_ was better than L64 and rL_3_PL_3_ [22,23]. However, we still do not know which is better, LPL or rL_2_PL_2_. Further, we need to make clear which kind of molecular structure is better, “hydrophobic-hydrophilic-hydrophobic” or “hydrophilic-hydrophobic-hydrophilic”. This is of special importance for further intramuscular gene delivery material design and optimization. When the DNA molecule encountered the LPL molecule in the membrane, the DNA transmembrane energy barrier would significantly increase again (Figure 11E), which made it difficult for DNA to penetrate the membrane. So, if the material molecules found it easier to leave the cell membrane than to remain within, there should be less opportunity for the DNA molecules to encounter the material molecules. However, the free energy of the material molecules was found to rise again when the molecules left the membrane (Figure 6A), indicating that there is resistance to them leaving the membrane. So, if a material molecule had a lower free energy change while exiting the membrane, it would be easier for the material molecule to leave the membrane. Here, it was clear that LPL had an obviously lower free energy change than rL_2_PL_2_ (Figure 6A) while exiting the membrane, so, LPL found it easier than rL_2_PL_2_ to leave the membrane. Moreover, as LPL was found to embed in a relatively shallow position in the cell membrane (Figure 3A), it was able to produce a disturbance effect faster than rL_2_PL_2_. In addition, for the “hydrophobic-hydrophilic-hydrophobic” copolymer of LPL, whether it was “LPL in” or “LPL on” state, the perturbation effect was largely the same (Figure 8A,B). The number of contacted phospholipid atoms had little effect on the perturbation effect. Considering that LPL has an opposite molecular structure to that of the cell membrane and LPL finds it easier to leave the membrane, we speculate that LPL molecules are more likely to pass through the cell membrane, i.e., the disturbance results mainly rely on the transmembrane action. This is quite different from the case with “hydrophilic-hydrophobic-hydrophilic” molecules, which mainly depend on the intermembrane perturbation effect via interaction with contacted phospholipid atoms.

## 5. Conclusions

In this study, we revealed the details of several typical and efficient intramuscular gene delivery materials at the atomic level, including the process involved in the material molecules entering and exiting the phospholipid membrane, interactions between material molecules and the membrane, interactions between material molecules and DNA molecules, and also including the free energy changes in the materials, and the changes in the transmembrane energy barrier to DNA molecules. More importantly, the simulation results almost completely matched our previous experimental results, thus proving the rationality of the molecular design of these materials. Regardless of whether the structure was “hydrophilic-hydrophobic-hydrophilic” or “hydrophobic-hydrophilic-hydrophobic”, the material molecules approached the cell membrane with their hydrophilic ends at first and then gradually entered the interior of the membrane through the interaction between their hydrophobic segments with the hydrophobic region of the membrane. They had no adverse effect on the fluidity of the cell membranes, indicating their biocompatibility with skeletal muscle cells. By analyzing the energy change, the spontaneity of the material molecules entering the cell membrane was proved. Upon the perturbation effects of material molecules, the transmembrane free energy barrier of DNA was decreased, making it easier for DNA to pass through the cell membrane. The simulation results also showed that there was little-to-no interaction between DNA and material molecules.

In addition, this study revealed and explained that the “hydrophobic-hydrophilic-hydrophobic” structure of LPL had several advantages over the “hydrophilic-hydrophobic-hydrophilic” structure of rL_2_PL_2_. The results of this study will help us to design and optimize intramuscular gene delivery materials.

## Figures and Tables

**Table 1:** Experimental parameters of gene delivery polymers.

	Molar Mass	Critical Micelle Concentration (mg/mL)	Work Concentration (wt%)
L64	2900	0.43	0.1
LPL	3775	0.089	0.0075
rL_2_PL_2_	4025	0.056	0.005
rL_3_PL_3_	5980	0.251	0.02

## Data Availability

All the data used in this article has been presented in the main text and Supporting Information.

## Supplementary Materials

The following supporting information can be downloaded at: https://www.mdpi.com/article/10.3390/jfb14040219/s1, Table S1: Details of the various systems simulated in this study; Figure S1: Initial model of four-groups; (A) System A, B, D, E; (B) System F-J; (C) System K-N; (D) System O-R; Figure S2: The process of L64 entering the phospholipid membrane (the model of 2 membranes); Figure S3: Final conformations of four molecules in PMF across phospholipid membranes: (a) L64; (b)LPL; (c) rL_2_PL_2_; (d) rL_3_PL_3_.
