# Supplementary material for: The Interaction Mechanism of Intramuscular Gene Delivery Materials with Cell Membranes
Source: J Funct Biomater. 2023 Apr 14;14(4):219. doi: 10.3390/jfb14040219 (PMC10144004; doi:10.3390/jfb14040219)
Supplement: Supplementary file 1 [file jfb-14-00219-s001.zip › jfb-2257905-supplementary.pdf]

Supporting information

The Interaction Mechanism of Intramuscular Gene Delivery Materials with Cell Membranes

Zhanpeng Cui [1], Yang Jiao [1], Linyu Pu [2], Jianlin Chen [3], Ming Liu [4], James Zhenggui Tang [5] and Gang Wang [1,*]

[1] National Engineering Research Center for Biomaterials, College of Biomedical Engineering,
Sichuan University, Chengdu 610064, China; 2019226075046@stu.scu.edu.cn (Z.C.);
jiaoyang@stu.scu.edu.cn (Y.J.)

[2] School of Materials and Chemistry, Southwest University of Science and Technology,
Mianyang 621010, China; linyu.pu@swust.edu.cn

[3] School of Laboratory Medicine, Chengdu Medical College, Chengdu 610500, China; jianlin1126@163.com

[4] Department of Medical Oncology/Gastric Cancer Center, West China Hospital, Sichuan University, Chengdu 610041, China; liuming629@wchscu.cn

[5] Research Institute of Healthcare Science, Faculty of Science & Engineering, University of Wolverhampton, Wolverhampton WV11SB, UK; j.z.tang@wlv.ac.uk

* Correspondence: wgang@scu.edu.cn

Table S1. Details of the various systems simulated in this study.

System label	Box size (nm³)	Water quantities	Description
**A**	9.0 × 9.0 × 16.0	29794	L64 & 240 Lipids
**B**	9.0 × 9.0 × 16.0	29794	LPL & 240 Lipids
**C**	9.0 × 9.0 × 16.0	29794	LPL & 240 Lipids (LPL in the Lipids membrane)
**D**	9.0 × 9.0 × 16.0	29794	rL_2PL_2 & 240 Lipids
**E**	9.0 × 9.0 × 16.0	29794	rL_3PL_3 & 240 Lipids
**F**	9.0 × 9.0 × 16.0	29764	PMF: L64 & 240 Lipids
**G**	9.0 × 9.0 × 16.0	29764	PMF: LPL & 240 Lipids
**H**	9.0 × 9.0 × 16.0	29764	PMF: rL_2PL_2 & 240 Lipids
**I**	9.0 × 9.0 × 16.0	29764	PMF: rL_3PL_3 & 240 Lipids
**J**	9.0 × 9.0 × 16.0	29764	PMF: rL_3PL_3-2 & 240 Lipids (Hydrophilic parts face down)
**K**	14.0 × 8.0 × 8.0	28958	L64 & DNA (No Lipids)
**L**	14.0 × 8.0 × 8.0	28958	LPL & DNA (No Lipids)
**M**	14.0 × 8.0 × 8.0	28958	rL_2PL_2 & DNA (No Lipids)
**N**	14.0 × 8.0 × 8.0	28958	rL_3PL_3 & DNA (No Lipids)
**O**	9.0 × 9.0 × 16.0	29766	PMF: DNA & 240 Lipids (None)
**P**	9.0 × 9.0 × 16.0	29766	PMF: DNA & 240 Lipids (L64)
**Q**	9.0 × 9.0 × 16.0	29766	PMF: DNA & 240 Lipids (DNA met the LPL)
**R**	9.0 × 9.0 × 16.0	29766	PMF: DNA & 240 Lipids (LPL)
**S**	8.5 × 8.5 × 18.9	25938	L64 & 240 Lipids (2 membranes)
**T**	7.1 × 7.1 × 7.8	6368	LPL & 160 Lipids
**U**	7.1 × 7.1 × 7.8	6368	rL_2PL_2 & 160 Lipids

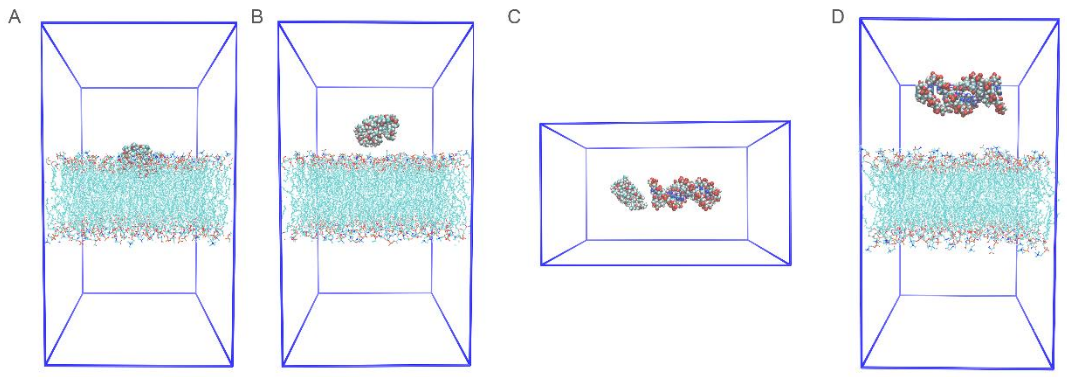

Figure. S1. Initial model of four-groups. (A)System A,B,D,E;(B)System F-J;(C) System K-N; (D) System O-R;

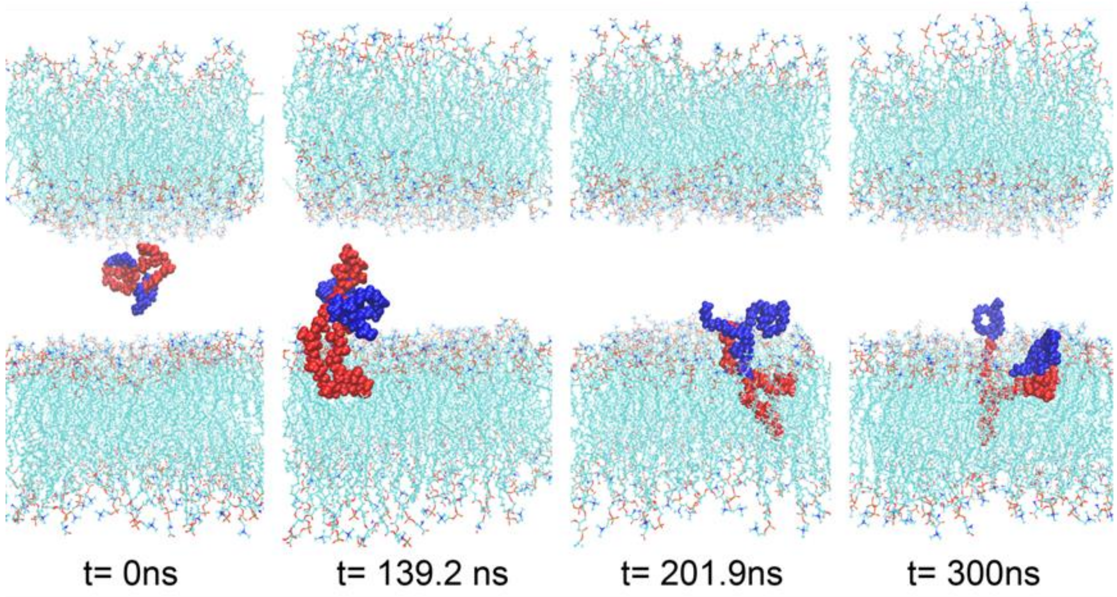

Figure. S2. The process of L64 entering the phospholipid membrane (the model of 2 membranes).

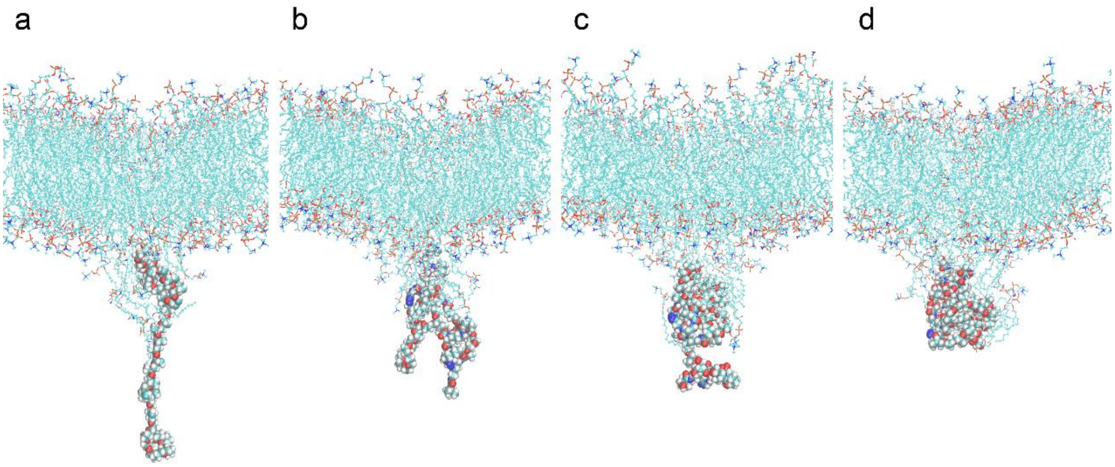

Figure. S3 Final conformations of four molecules in PMF across phospholipid membranes: (a) L64; (b)LPL; (c) rL_2PL_2; (d) rL_3PL_3.
